# Detection of Infectious Laryngotracheitis Virus by Real-Time PCR in Naturally and Experimentally Infected Chickens

**DOI:** 10.1371/journal.pone.0067598

**Published:** 2013-06-28

**Authors:** Yan Zhao, Congcong Kong, Xianlan Cui, Hongyu Cui, Xingming Shi, Xiaomin Zhang, Shunlei Hu, Lianwei Hao, Yunfeng Wang

**Affiliations:** 1 Division of Avian Infectious Diseases, State Key Laboratory of Veterinary Biotechnology, Harbin Veterinary Research Institute, The Chinese Academy of Agricultural Sciences, Harbin, China; 2 National Engineering Research Center of Veterinary Biologics, Harbin, China; 3 Animal Health Laboratory, Department of Primary Industries, Parks, Water and Environment, Tasmania, Australia; 4 Institute of Animal Science and Technology, Yunnan Agricultural University, Kunming, China; NIAID, United States of America

## Abstract

Infectious laryngotracheitis (ILT) is an acute, highly contagious upper-respiratory infectious disease of chickens. In this study, a real-time PCR method was developed for fast and accurate detection and quantitation of ILTV DNA of chickens experimentally infected with ILTV strain LJS09 and naturally infected chickens. The detection lower limit of the assay was 10 copies of DNA. There were no cross reactions with the DNA and RNA of infectious bursal disease virus, chicken anemia virus, reticuloendotheliosis virus, avian reovirus, Newcastle disease virus, and Marek's disease virus. The real-time PCR was reproducible as the coefficients of variation of reproducibility of the intra-assay and the inter-assay were less than 2%. The real-time PCR was used to detect the levels of the ILTV DNA in the tissues of specific pathogen free (SPF) chickens infected with ILTV at different times post infection. ILTV DNA was detected by real-time PCR in the heart, liver, spleen, lung, kidney, larynx, tongue, thymus, glandular stomach, duodenum, pancreatic gland, small intestine, large intestine, cecum, cecal tonsil, bursa of Fabricius, and brain of chickens in the infection group and the contact-exposure group. The sensitivity, specificity, and reproducibility of the ILTV real-time PCR assay revealed its suitability for detection and quantitation of ILTV in the samples from clinically and experimentally ILTV infected chickens.

## Introduction

Infectious laryngotracheitis (ILT) is an acute, highly contagious upper-respiratory infectious disease of chickens, which was first described in the USA in 1925 [1]. The symptoms of ILT are nasal discharge, conjunctivitis, reduced egg production, gasping, coughing, expectoration of bloody mucus, and marked dyspnea that may lead to suffocation. This disease causes economic losses in poultry industries worldwide [2]. Gallid herpesvirus 1, which is also called infectious laryngotracheitis virus (ILTV), is a member of the family Herpesviridae according to the Ninth International Committee on the Taxonomy of Viruses (ICTV) [3]. Although virus isolation has been used to detect ILTV, it is time consuming. Serological tests, including fluorescent antibody technique (FAT) [4], indirect immunofluorescence (IIF) [5], conventional enzyme-linked immunosorbent assay (ELISA) [6], serum neutralization (SN) [7], and agar gel immunodiffusion (AGID), have also been used, but they are generally of low sensitivity and laborious. DNA detection by conventional polymerase chain reaction (PCR) or real-time PCR has become a preferred method of virus diagnosis [8], [9], [10], [11]. Real-time PCR has gained wide acceptance due to its improved rapidity, sensitivity, reproducibility, and the reduced risk of carryover contamination [12].

In this study, a rapid and sensitive real-time PCR assay for detection and quantitation of ILTV was developed and evaluated. The sensitivity, specificity, and reproducibility of the ILTV real-time PCR assay were proven to be suitable for detection and quantitation of ILTV DNA. By using this technique, the levels of ILTV DNA in the tissues of the chickens infected with ILTV at different times and in the clinical cases were determined and quantified.

## Materials and Methods

### Ethics Statement

Animal experiments were approved by Animal Ethics Committee of Harbin Veterinary Research Institute of the Chinese Academy of Agricultural Sciences (CAAS) and performed in accordance with animal ethics guidelines and approved protocols. The Animal Ethics Committee approval number was SYXK (Hei) 2011022.

### Virus strains

The ILTV strain LJS09 was stored at Harbin Veterinary Research Institute of CAAS. Infectious bursal disease virus (IBDV), chicken anemia virus (CAV), reticuloendotheliosis virus (REV), avian reovirus (ARV), Newcastle disease virus (NDV), and Marek's disease virus (MDV) were provided by other avian infectious disease laboratories of Harbin Veterinary Research Institute of CAAS.

### Virus isolation and identification

ILTV LJS09 strain was isolated in 2009 from six-week-old Yellow-Foot layers on a farm in Jiangsu Province in China. These chickens were not vaccinated against ILT, and they experienced gasping and fits of coughing before death. Larynx tissues and caseous exudates were collected. All of the specimens were homogenized, freeze-thawed three times, and treated with penicillin (500 IU/ml) and streptomycin (500 IU/ml). The specimens were inoculated onto the chorioallantoic membranes (CAMs) of 9-day-old specific-pathogen-free (SPF) chicken embryos, which were incubated at 37°C and examined daily for 5 days, after which the CAMs and allantoic fluids were harvested, homogenized, and freeze-thawed three times. The specimens were inoculated on chicken embryo kidney (CEK) cells, and 48 h post infection the CEK cells were treated by fixation, dehydration, embedding, polymerization, and then cut into 50 nm sections, which were stained by 1% uranyl acetate and 1% lead citrate for transmission electron microscopy (Hitachi H-7650, Japan). Meanwhile, DNA was extracted from the specimen infected CEK cells for PCR analysis using a pair of gB Forward/Reverse primers designed based on the ILTV gB gene.

### Virus propagation and genome sequence determination

The ILTV strain LJS09 was inoculated onto the CAMs of 9-day-old SPF chicken embryos as described previously [13], [14], which were incubated at 37°C and examined daily for 5 days until the CAMs and allantoic fluids were harvested. The virus titer was titrated by inoculation onto the CAMs of at least six different dilutions of the inocula (four eggs per dilution). The inoculated eggs were examined on the fifth day post inoculation and the titer was calculated using the method described by Reed and Muench [15].

Genomic DNA of ILTV was extracted according to the manufacturer's instructions of TIANamp Genomic DNA Kit (TIANGEN, Beijing, China). The genome of LJS09 strain was amplified by conventional PCR, and a total of 102 pairs of primers (data not shown) were designed according to ILTV strain Serva (accession No. HQ630064) and pairs of the neighboring primers were overlapped. DNA sequences were assembled using the Seqman program (DNASTAR, Madison, WI). The complete sequence of ILTV LJS09 was deposited at GenBank.

### Preparation of genomic DNA and RNA of reference viruses

Genomic DNA and RNA from reference viruses including IBDV, CAV, REV, ARV, NDV and MDV were extracted using TIANamp Genomic DNA Kit (TIANGEN, Beijing, China) and Simply P Total RNA Extraction Kit (BioFlux, Hangzhou, China) according to the manufacturer's instructions.

### PCR primers and the probe

A pair of primers (gB-S: 5′ CAGTATCTGGCATCGCCTCAT 3′; gB-A: 5′ CCTGGGAACAGAACCTGAACT 3′) and the probe (5′ FAM-CTAACCCGTTCG CCGCACTCG-BHQ-1 3′) for real-time PCR were designed based on a highly conserved region of the gB gene (GenBank accession no: EU104985) to amplify a 148 bp fragment. Conventional PCR primers (gB Forward: 5′ TTCCGAGATCGAAGAAGTGAG 3′; gB Reverse: 5′ ACTCTGGTGGCAAGTATCCTGT 3′) were designed according to the conserved region of the gB gene (GenBank accession no: EU104985) to amplify a 567 bp fragment. Primers and the probe were designed using the Primer Premier software (version 5.0). All primers and probe were synthesized by Invitrogen (Beijing, China).

### Preparation of standard DNA solutions

A 148 bp fragment of the gB gene was amplified by PCR from DNA of ILTV strain LJS09 with gB-S and gB-A primers, cloned into pMD18-T vector (TaKaRa, Dalian, China), and transformed into *E. coli* DH5α competent cells. The recombinant plasmid DNA was extracted using Biospin plasmid DNA extraction kit (BioFlux, Hangzhou, China) and confirmed by sequencing, which was named pT-gB. The pT-gB DNA concentration was determined by spectrophotometry at 260 nm and the purity was confirmed using the 260/280 ratio. The copy number of plasmid was calculated using the equation: Plasmid copies/µl = [plasmid amount (g/µl)×(6.02×10^23^)]/[plasmid length (bp)×660]. The serial dilutions of the standard DNA were used for all quantitation assays.

### Conventional PCR

The 25 µl PCR reactions contained 2.5 µl of 10× PCR buffer (Mg^2+^ Plus) (TaKaRa, Dalian, China), 2 µl of dNTP (2.5 mM each), 0.2 µl of Taq DNA polymerase (5 U/µl) (TaKaRa, Dalian, China), 1 µl of each primer (gB Forward/Reverse primers) (10 pmol/µl), and 1 µl of DNA template (7.75×10^−2^ ng/µl to 7.75×10^−8^ ng/µl; 7.75 ng equals 2.5×10^9^ copies). The reaction mixture was incubated at 95°C for 3 min, then subjected to 40 cycles of 94°C for 10 s, 60°C for 20 s and 72°C for 20 s, and finally incubated at 72°C for 5 min. The PCR products were analyzed by electrophoresis.

### Optimization of the real-time PCR

The real-time PCR was performed using the LightCyclerR480 (Roche Diagnostics GmbH, Mannheim, Germany). The PCR conditions, including the concentrations of primers and the probe, were optimized to obtain optimal specific fluorescent signals. The real-time PCR was carried out in a total volume of 20 µl containing 10.0 µl of 2×Premix Ex Taq (TaKaRa, Dalian, China), 0.4 µl of each primer (2.5, 5, 10, 15, and 20 pmol/µl) (gB-S/A), 0.8 µl of the probe (2.5, 5, 10, 15, and 20 pmol/µl), 2 µl of DNA template (3.1×10^−2^ ng/µl to 3.1×10^−8^ ng/µl; 3.1 ng equals 10^9^ copies), and 6.4 µl of ddH_2_O. The reaction was carried out with a pre-denaturation at 95°C for 30 s, followed by 40 cycles of denaturation at 95°C for 5 s and annealing/elongation at 60°C for 20 s. The fluorescent signals were measured at the end of the annealing/elongation step. Negative control was set up by substituting the DNA template with ddH_2_O. Conditions were selected to ensure that Ct values were the lowest possible and the fluorescence acquisition curves were robust to each DNA concentration. In each run, a series of dilutions of the standard plasmid DNA were also included along with DNA samples. The quantitation data, in terms of Ct values, were determined using the Abs Quant/Fit Points of the LightCycler software, version 1.5.0.39 (Roche Diagnostics GmbH, Mannheim, Germany).

### Establishment of the real-time PCR standard curve

Serial dilutions of recombinant pT-gB plasmid DNA ranging from 10^7^ copies/µl to 10^1^ copies/µl were made in sterile ddH_2_O. To generate standard curves, the dilutions were tested in triplicate and used as quantification standards to construct the standard curve by plotting the plasmid copy number logarithm against the measured Ct values. The LightCycler software was used to generate the standard curve and to calculate the correlation coefficient of the standard curve.

### Sensitivity, specificity, and reproducibility tests

The sensitivity of the real-time PCR was determined by using serially diluted recombinant pT-gB plasmid DNA from 10^7^ copies/µl to 10^1^ copies/µl under the optimized conditions, and compared with conventional PCR. The detection limit of the conventional PCR was determined based on the DNA copies with a positive result producing a 148 bp fragment on agarose gel. The detection limit of the real-time PCR was determined based on the highest dilution with the presence of a Ct value.

To evaluate the specificity of the real-time PCR for ILTV detection, DNA and RNA samples from other avian viruses, including IBDV, CAV, REV, ARV, NDV, and MDV, were tested with DNA template of ILTV strain LJS09 as positive control and ddH_2_O as negative control.

To evaluate the reproducibility of the real-time PCR, intra-assay and inter-assay tests were performed in triplicates by testing four dilutions of DNA samples extracted from ILTV strain LJS09 within the same run and independently in three different runs. All the tests were performed three times. The mean of CT values and the coefficient of variation (CV) values were evaluated.

### Detection of clinical samples from the field

Ten clinical samples were collected from six provinces in China from 2011 to 2012. All the samples were homogenized, freeze-thawed three times, centrifugated, and then inoculated onto the CAMs of 9-day-old SPF chicken embryos for virus isolation and PCR identification as described previously. DNA of the ten isolates was extracted from the supernatant of the allantoic fluid and CAM homogenates. All the samples were tested in triple by real-time PCR. The information of the ten samples is listed in Table 1.

**Table 1:** Isolation and identification of ILTV in ten clinical samples.

Isolation date	Name	Type of chickens	History of ILT vaccination	Tissue	Age of chicken	Mean Ct ± S.D.
Apr 2011	MDJ0442	Layers	Unvaccinated	Larynx	130-day-old	30.39±0.27
May 2011	HLJ0507	Layers	Unvaccinated	Larynx	100-day-old	30.85±0.10
Oct 2011	SY1014	Layers	Unvaccinated	Larynx and trachea	146-day-old	28.31±0.09
Jan 2012	SD1244	No record	No record	Kidney	No record	33.13±0.33
Jan 2012	HB1229	No record	No record	Trachea	No record	28.92±0.06
Jan 2012	ZHJ1298	No record	No record	Trachea	No record	31.62±0.47
Feb 2012	SD0203	Layers	Vaccinated against ILT CEO vaccine	Larynx and trachea	240-day-old	27.59±0.26
Mar 2012	JS12-9	Layers	Vaccinated against ILT CEO vaccine	Larynx	400-day-old	33.97±0.02
Mar 2012	JS12-4	Layers	Unvaccinated	Larynx	70-day-old	32.60±0.15
Dec 2012	MS009	Layers	Vaccinated against ILT CEO vaccine	Larynx	300-day-old	28.70±0.20

### Experimental infection of SPF chickens and preparation of tissue samples

Fifty 10-week-old SPF chickens from the Experimental Animal Center of Harbin Veterinary Research Institute of CAAS, China, were randomly divided into three groups and raised in two negative pressure isolators. In brief, 25 chickens were inoculated intratracheally with 10^4^ EID_50_ of ILTV strain LJS09 (named infection group), and another 15 raised in the same negative pressure isolator (named contact-exposure group). Ten chickens were raised in a separate negative pressure isolator (named control group). Clinical symptoms were observed daily. Three chickens from the infection group, two chickens from the contact-exposure group, and one from the control group were euthanized at each time point. DNA samples extracted from heart, liver, spleen, lung, kidney, larynx, tongue, thymus, glandular stomach, duodenum, pancreatic gland, small intestine, large intestine, cecal tonsil, bursa of Fabricius, and brain were analyzed by the real-time PCR at 1, 3, 7, 14 and 28 days post-inoculation (dpi). The tissue samples were collected from the chickens and frozen at −80°C. One gram of each tissue specimens was weighed, added into 1 ml of phosphate buffered saline (PBS, pH 7.4) and homogenized. DNA was extracted from 200 µl of homogenized tissue samples by using TIANamp Genomic DNA Kit (TIANGEN, Beijing, China), and eluted in 50 of µl ddH_2_O. The viral load was quantified by real-time PCR using 2 µl of DNA. All the samples were tested in triplicates. The virus concentrations were expressed as the mean virus genome copy numbers in 1 g of the tested specimens.

### ILTV immunohistochemistry

All the tissues from the infection group, the contact-exposure group and the control group were collected at each observation time point. ILTV immunohistochemistry was performed on 4 µm thick sections of formalin-fixed, paraffin-embedded (FFPE) tissue. Tissue sections were blocked with 5% BSA at 37°C for 30 min and incubated with 1∶10 diluted ILTV primary polyclonal antibodies at 4°C over-night. Tissue sections were then incubated with a biotin-conjugated goat anti-chicken IgY secondary antibody (Santa Cruz Biotechnology Inc., Santa Cruz, CA). Antibody binding was visualized with the strept avidin-biotin complex (SABC) (BOSTER, Wuhan, China). Staining was performed with DAB and counterstaining with hemalum.

## Results

### Isolation and identification of ILTV

**Figure 1:**
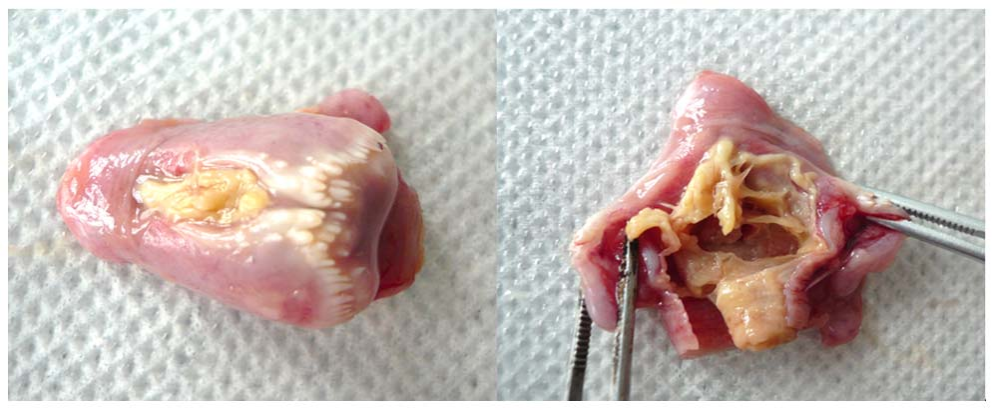
Pathological anatomy of the larynx of ILTV infected chicken. Larynx of the ILTV infected chicken was blocked by yellow caseous clots. Cutting the larynx open, the lumen of the larynx was narrow and the whole lumen was coated with yellow caseous membrane.

**Figure 2:**
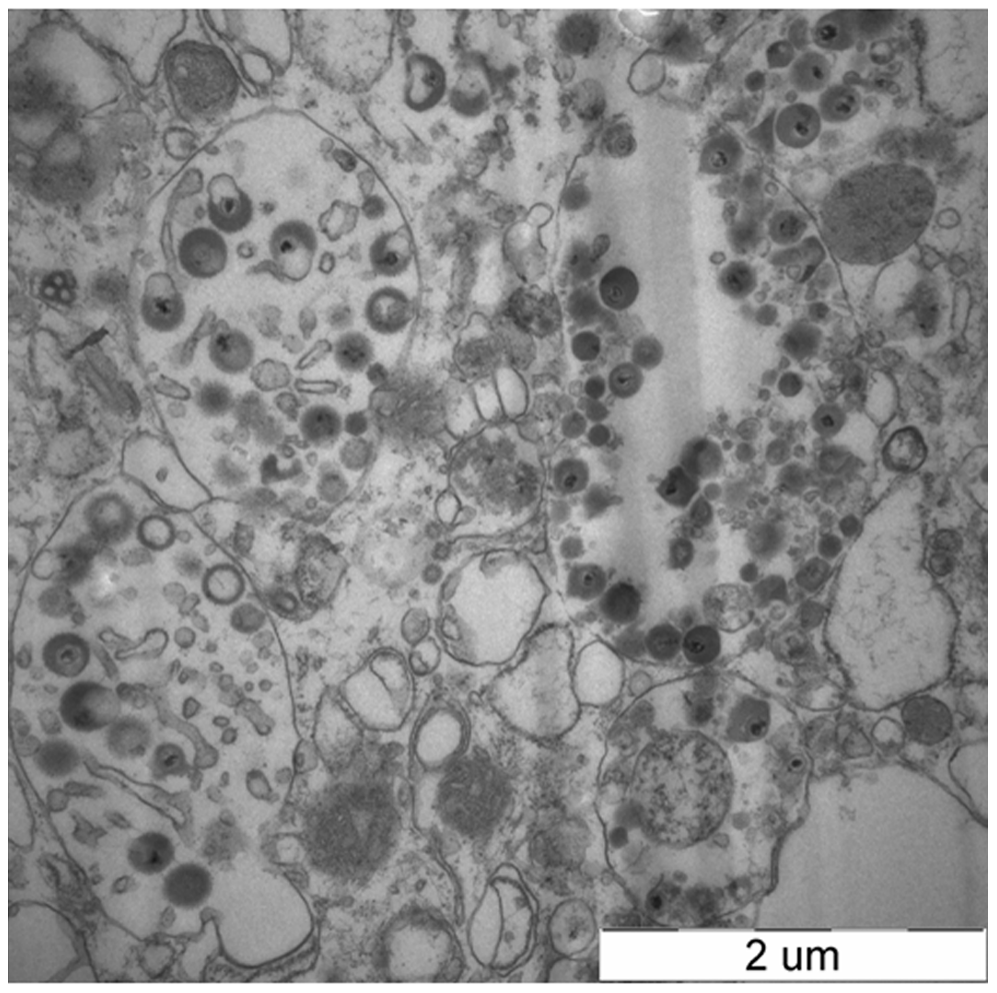
The transmission electron microscopy of ILTV virions in chicken embryo kidney cells. The chicken embryo kidney cells inoculated with the ILTV specimens for 48 h were treated by fixation, dehydration, embedding, polymerization, cutting into 50 nm sections, and then stained by 1% uranyl acetate and 1% lead citrate for transmission electron microscopy. The ILTV virions were distributed in cytoplasm of the chicken embryo kidney cells. The scale at the bottom-right of the picture was 2 µm.

Larynx tissue of the ILTV infected chicken was full of caseous exudates (Fig. 1), which was in agreement with the clinical signs before death. CAMs of SPF chicken embryos were examined on the fifth day post inoculation. The infected CAMs were thickened and showed white opaque plaques. Severe hyperemia appeared in the infected embryos. The electron microscopy of the chicken embryo kidney cells inoculated with the specimens showed that the ILTV virions were distributed in cytoplasm (Fig. 2). The PCR product amplified using the gB primers with the genomic DNA templates was 567 bp in length (data not shown).

### Genome sequence determination

The genome size of ILTV LJS09 was 153,013 bp, with a G+C content of 48.1%. The genome sequence of ILTV strain LJS09 has been deposited in the GenBank (accession no. JX458822).

### Optimization of real-time PCR and establishment of the standard curve

The concentration of recombinant pT-gB plasmid DNA was 60.25 ng/µl and the A260/A280 ratio was 1.85. The conversion copy number of pT-gB plasmid DNA was 1.93×10^10^ copies/µl. After optimization, the final total amount of each primer was 4 pmol and the probe was 8 pmol. In brief, the real-time PCR in a 20 µl reaction volume contained 10.0 µl of 2× Premix Ex Taq, 0.4 µl of each primer (10 pmol/µl) (gB-S/A), and 0.8 µl of the probe (10 pmol/µl), 2 µl of DNA template (3.1×10^−2^ ng/µl to 3.1×10^−8^ ng/µl; 3.1 ng equals10^9^ copies), and 6.4 µl of ddH_2_O.

**Figure 3:**
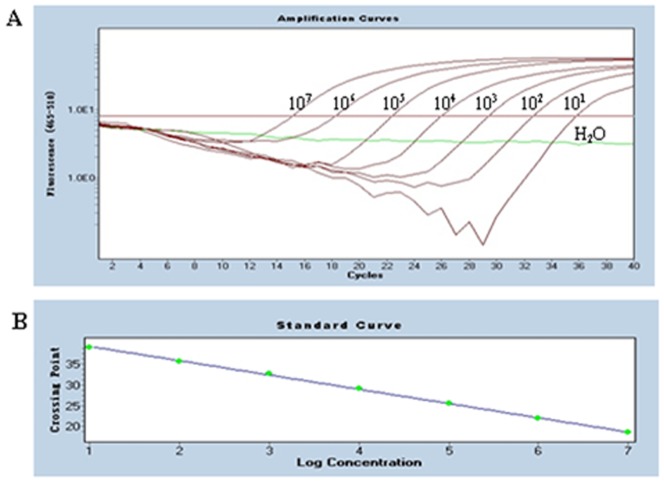
Amplification curve and standard curve of gB assay. A: Amplification curves. Ten-fold dilutions of standard DNA ranging from 10^7^ copies/µL to 10^1^ copies/µL were used as standard controls. B: Standard curve (Y = −3.442X+42.76) (Error = 0.0654 and Efficiency = 1.952) was analyzed with the LightCycler 480 software 1.5.0.39. The concentration refers to the template copy number per reaction. Standards of DNA are indicated on the x-axis whereas the corresponding cycle threshold (Ct) values are presented on the y-axis.

The real-time PCR amplification curves and standard curves were generated by employing the serially diluted pT-gB plasmid DNA from 10^7^ to 10^1^ copies/µl and detected by real-time PCR under the optimized conditions (Fig. 3A). The threshold cycle (Ct) values were then plotted against the known copy number of the standard controls. The error value (0.0654) was below 0.2 (Fig. 3B), which was acceptable according to the LightCycler 480's instructions. The efficiency was 1.952, the slope was −3.442, and the Y intercept was 42.76. We quantified the amount of unknown samples by using the following formula: Y = −3.442X+42.76 (Y = threshold cycle, X = log starting quantity).

### Sensitivity, specificity, and reproducibility of the real-time PCR

**Figure 4:**
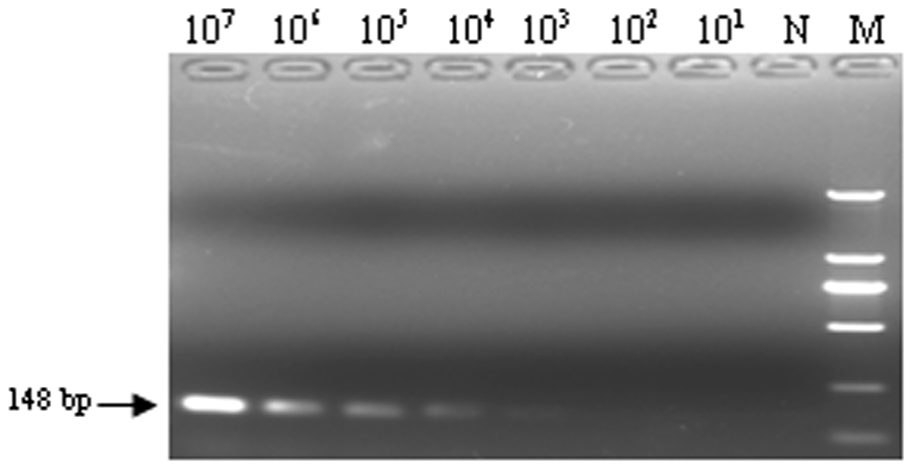
Sensitivity of the conventional PCR. Ten-fold dilutions of standard DNA ranging from 10^7^ copies/µL to 10^1^ copies/µL were used to determine the sensitivity of the conventional PCR. The PCR products were stained with ethidium bromide. N: H_2_O; M: DL2000 DNA marker.

Serial dilutions of the recombinant pT-gB plasmid DNA ranging from 10^7^ to 10^1^ copies/µl were tested by the established real-time PCR to evaluate the sensitivity. The lower detection limit was 10 copies per reaction (Fig. 3A) whereas it was 10^3^ copies of DNA for conventional PCR (Fig. 4). These results indicated that the real-time PCR was 100 times more sensitive than the conventional PCR.

**Figure 5:**
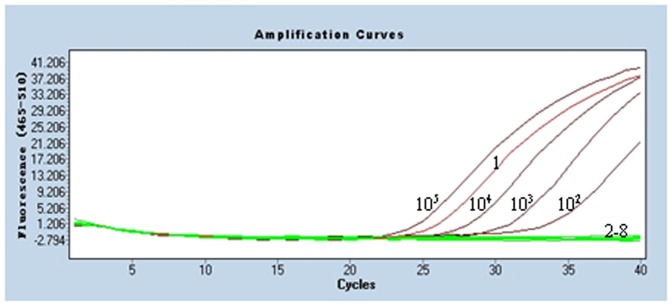
Specificity of the real-time PCR. Six other avian pathogens were used for the specificity test. Dilutions of 10^5^, 10^4^, 10^3^, 10^2^ were the standard DNA; 1: ILTV LJS09 strain DNA; 2–7: DNA and RNA samples of IBDV, CAV, REV, ARV, NDV, MDV; 8: H_2_O.

The specificity of real-time PCR was tested using the standard plasmid pT-gB DNA, DNA and RNA samples extracted from ILTV, IBDV, CAV, REV, ARV, NDV and MDV as well as a blank control with water. The gB-specific probe detected the ILTV strain LJS09 and the plasmid standard, but no detectable fluorescent signals were observed for other avian viruses and the blank control sample (Fig. 5), suggesting that the established real-time PCR assay was highly specific.

The reproducibility of real-time PCR for detection of DNA extracted from 10-fold serially diluted virus suspensions of ILTV strain LJS09 was determined three times for both intra-assays and inter-assays. The mean intra-assay and inter-assay coefficients of variation (CVs) were both below 2%. The intra-assay CVs were 0.51–1.28% while inter-assay CVs were 0.73–1.68% (Table 2).

**Table 2:** Reproducibility of real-time PCR.

Dilution of virus	Repeats	Reproducibility of intra-assay		Reproducibility of inter-assay	
		Mean Ct±S.D.	CV(%)	Mean Ct±S.D.	CV(%)
10^0^	3	24.86±0.14	0.58	25.06±0.29	1.18
10^−1^	3	26.55±0.33	1.28	27.05±0.45	1.68
10^−2^	3	31.09±0.23	0.74	31.38±0.36	1.16
10^−3^	3	34.37±0.17	0.51	34.45±0.25	0.73

Note: Four dilutions of the ILTV LJS09 strain were detected in intra- assay and inter-assay tests. The coefficients of variation (CVs) of both tests were below 2%.

### Detection of clinical samples from the field

DNA extracted from the clinical samples of ILTV infected chickens was detected by real-time PCR. All of the ten samples were ILTV positive. The Ct values of these samples were from 27 to 34 (Table 1).

### Tissue distribution of ILTV as detected by real-time PCR

DNA of ILTV in the tissues of chickens was determined by using the established real-time PCR, and the DNA copy number of each sample was converted to copy number per gram by using the calculated Ct value determined from the standard curve. The dynamic distribution of ILTV in the tissues from the infection group, the contact-exposure group, and the control group were determined by the copy numbers. The detection ratios of ILTV DNA in each tissue at different days post infection are shown in Table 3. All the tissues in the infection group and the contact-exposure group, including the heart, liver, spleen, lung, kidney, larynx, tongue, thymus, glandular stomach, duodenum, pancreatic gland, small intestine, large intestine, cecal tonsil, bursa of Fabricius and brain, were positive at 3, 7, and 14 dpi. However, the positive detection ratios of individual tissues in the infection group at 1 and 28 dpi were between 33–66%, while all the tissues were negative in the contact-exposure group at 1 dpi.

**Table 3:** Distribution of ILTV DNA in the tissues of infected chickens at different days post infection.

		Positive ratio of ILTV DNA at different days post infection				
Tissue	Group	1 d	3 d	7 d	14 d	28 d
Heart	I^a^	3/3	3/3	3/3	3/3	1/3
	C^b^	-/-C	2/2	2/2	2/2	2/2
Liver	I	3/3	3/3	3/3	3/3	3/3
	C	-/-	2/2	2/2	2/2	2/2
Spleen	I	3/3	3/3	3/3	3/3	1/3
	C	-/-	2/2	2/2	2/2	2/2
Lung	I	3/3	3/3	3/3	3/3	3/3
	C	-/-	2/2	2/2	2/2	2/2
Kidney	I	3/3	3/3	3/3	3/3	3/3
	C	-/-	2/2	2/2	2/2	2/2
Laryngotrachea	I	3/3	3/3	3/3	3/3	3/3
	C	-/-	2/2	2/2	2/2	2/2
Tongue	I	3/3	3/3	3/3	3/3	3/3
	C	-/-	2/2	2/2	2/2	1/2
Thymus	I	3/3	3/3	3/3	3/3	3/3
	C	-/-	2/2	2/2	2/2	2/2
Glandular stomach	I	1/3	3/3	3/3	3/3	3/3
	C	-/-	2/2	2/2	2/2	2/2
Duodenum	I	3/3	3/3	3/3	3/3	2/3
	C	-/-	2/2	2/2	2/2	2/2
Pancreatic gland	I	3/3	3/3	3/3	3/3	3/3
	C	-/-	2/2	2/2	2/2	2/2
Small intestine	I	3/3	3/3	3/3	3/3	3/3
	C	-/-	2/2	2/2	2/2	2/2
Large intestine	I	3/3	3/3	3/3	3/3	3/3
	C	-/-	2/2	2/2	2/2	2/2
Cecum	I	3/3	3/3	3/3	3/3	3/3
	C	-/-	2/2	2/2	2/2	1/2
Cecal tonsil	I	3/3	3/3	3/3	3/3	1/3
	C	-/-	2/2	2/2	2/2	1/2
Bursa of Fabricius	I	3/3	3/3	3/3	3/3	1/3
	C	-/-	2/2	2/2	2/2	2/2
Brain	I	3/3	3/3	3/3	3/3	3/3
	C	-/-	2/2	2/2	2/2	2/2

Notes: Ten-week-old SPF chickens were inoculated intratracheally with 10^4^ EID_50_ of ILTV strain LJS09. The tissues with positive detection rates of less than 100% in ILTV real-time PCR were underlined ; the tissues from the control group were all negative (not shown);

a : I = infection group;

b : C = contact exposure group;

c : -/- means that the DNA were not detected at that time point.

**Figure 6:**
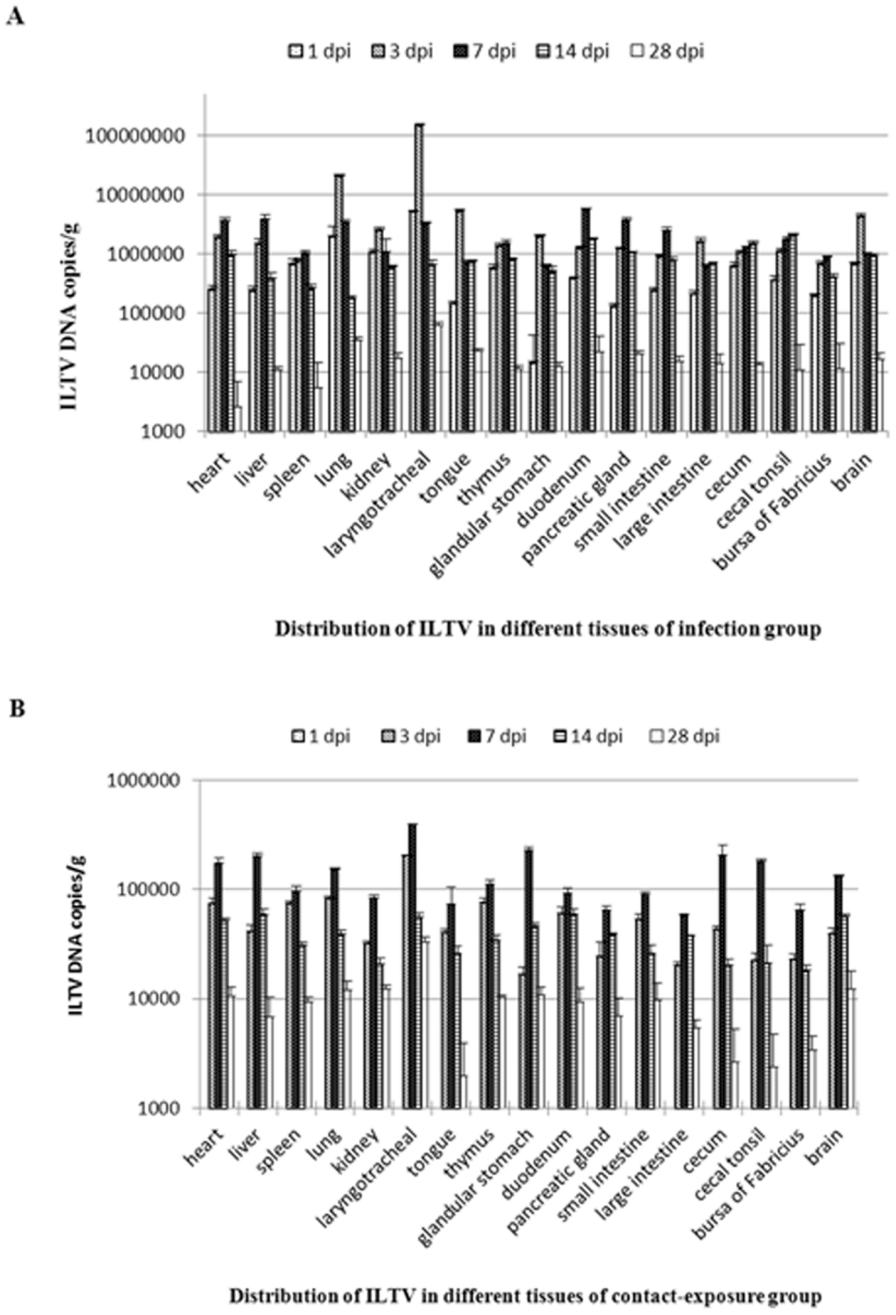
Distribution and quantity of ILTV DNA in each tissue of the chickens at different days post infection. Ten-week-old SPF chickens were inoculated intratracheally with 10^4^ EID_50_ of ILTV strain LJS09. The viral loads were given as ILTV DNA copy number per g in each tissue. The tissues included the heart, liver, spleen, lung, kidney, larynx, tongue, thymus, glandular stomach, duodenum, pancreatic gland, small intestine, large intestine, cecum, cecal tonsil, bursa of Fabricius, and brain. A: The mean viral load of three chickens in the infection group and the standard deviation for each time point. B: The mean viral load of two chickens in the contact exposure group and the standard deviation for each time point.

The copy numbers of ILTV DNA in each tissue from the infection group fluctuated between 10^3^–10^8^ copies/g at all observation points. The copy numbers of ILTV DNA of the majority of tissues peaked at 7 dpi, and the lung and larynx at 3 dpi had significantly higher copy numbers (10^7^–10^8^ copies/g) of ILTV DNA than the other tissues (10^5^–10^6^ copies/g). The copy numbers of ILTV DNA decreased from 14 dpi and the level of ILTV DNA decreased remarkably at 28 dpi (Fig. 6A). The fluctuant tendency of ILTV DNA in the contact-exposure group is shown in Fig. 6B. The copy numbers of ILTV DNA in each tissue peaked at 7 dpi, and then decreased from 14 dpi. The level of ILTV DNA in the contact-exposure group was 10 to 100 fold lower than that in the infection group at each observation point, except for the 28 dpi, at which the level of ILTV DNA in the two groups was almost the same (Fig. 6B). The chickens in the infection group presented clinical signs of ILT at 3–7 dpi, which experienced mucoid nasal discharges, gasping and fits of coughing. However, the clinical symptoms of chickens in the contact-exposure group were relatively moderate, and appeared 2 or 3 days late than the infected chickens. In addition, the chickens in the control group did not show any positive results at any time point or in any tissues (data not shown).

### Analysis of immunohistochemical staining

**Figure 7:**
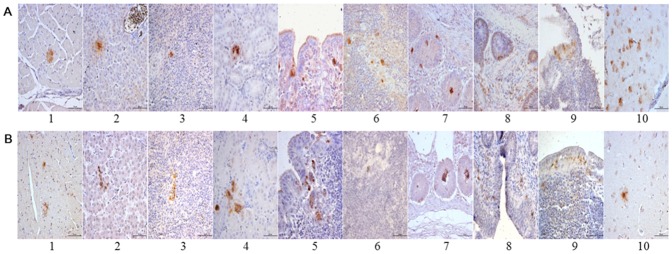
Immunohistochemical staining of the tissue sections at 14 dpi. A: Tissues from the infection group. B: Tissues from the contact-exposure group. The immunohistochemical staining results revealed that ILTV was detected in the tissues including the heart, liver, spleen, kidney, larynx, thymus, small intestine, large intestine, bursa of Fabricius, and brain in the infection group and the contact-exposure group. 1. Heart: ILTV resided among the cardiac muscle fiber; 2. Liver: ILTV resided in hepatocytes; 3. Spleen: ILTV resided in splenocytes; 4. Kidney: ILTV resided in renal tubular epithelial cells; 5. Larynx: ILTV resided under the mucous membrane of the larynx; 6. Thymus: ILTV resided in ovarian medulla; 7. Small intestine: ILTV resided in intestinal gland epithelial cells; 8. Large intestine: ILTV resided in cells of lamina propria inner layer, intestinal mucosa epithelial cells, and intestinal gland epithelial cells; 9. Bursa of Fabricius: ILTV resided in epithelial cells of bursa of Fabricius; 10. Brain: ILTV resided in cerebral cortex.

The immunohistochemical staining results showed that the majority of the tissues in the infection group and the contact-exposure group, including the heart, liver, spleen, kidney, larynx, thymus, small intestine, large intestine, bursa of Fabricius and brain, were positive at 3, 7, and 14 dpi (Fig. 7). Some tissues including the lung, tongue, glandular stomach, duodenum, pancreatic gland, cecum and cecal tonsil were negative (data not shown).

## Discussion

Viral isolation and serological tests have been used for ILTV diagnoses [4], [5], [6], [7]. However, these methods are laborious and time consuming and their sensitivities are low. Several PCR and real-time PCR techniques have been developed for ILTV detection [9], [10], [11], [16], [17]. Real-time PCR has become a potentially powerful technique in microbiological diagnostics because of its simplicity, rapidity, reproducibility and high sensitivity compared to other diagnostic methods. In this study, we report a real-time PCR assay for detection and quantification of ILTV DNA in ILTV infected chickens and in the clinical samples.

The genome sequence of ILTV is approximately 150 kb in length, consisting of 79 open reading frames [18], [19], [20]. Several highly conserved genes within the herpesviruses were selected for establishment of real-time PCR in previous studies, such as gG and TK genes [21], [22], [23] and the UL15a gene [11], [24], [25]. In this study, the sequences of gB genes of numerous ILTV strains from GenBank were analyzed and the similarity was 99.2–100%. Thus, a real-time PCR with high specificity and sensitivity for ILTV detection was developed based on a pair of primers and a specific probe designed according to the highly conserved region of the ILTV gB gene. The conditions of the real-time PCR were optimized to obtain highly specific fluorescent signals.

The standard curves in this study were generated using serially diluted pT-gB plasmids from 10^7^ copies/µL to 10^1^ copies/µL, which maintained linearity for seven orders of magnitude with an efficiency of 1.952 (the maximum efficiency is 2). The efficiency in this study was 97.6%, which was a little higher than those of two previous studies. The standard curve maintained linearity for at least five orders of magnitude with an overall efficiency of 94.54% in Callison's study [16] while the linearity was maintained for seven orders of magnitude with the average efficiency of 96.36% in Mahmoudian's study [11].

Real-time quantitative PCR assay usually offers a higher sensitivity than conventional PCR and the post PCR processing step is avoided, allowing savings in both time and reagents. Sensitive detection and quantification of ILTV DNA is pivotal *in vitro* and *in vivo* experiments. The lower detection limit of real-time PCR was 10 copies per reaction, which is ten times more sensitive than those of the previous studies [11], [16] and 100 times more sensitive than conventional PCR. In addition, the real-time PCR assay permits the simultaneous detection and quantification of DNA, and it requires less time to perform and provides a more objective final analysis as compared to the conventional PCR. It is also useful for understanding the mechanisms of virus transmission by investigating the viral dynamics [12]. The high specificity of this technique was confirmed by the negative detection signals for DNA and RNA of other avian viruses, such as IBDV, CAV, REV, ARV, NDV, and MDV. It showed high reproducibility with less than 2% intra-assay and inter-assay variability.

The real-time PCR assay was also found to be highly reproducible throughout the study and was able to determine the level of viral DNA in different tissues of the ILTV infected chickens at different times post infection. The results from the experimental infection showed that the ILTV DNA in all of the seventeen tissues from the infection group and the contact-exposure group were positive at 3, 7, 14 dpi. The level of ILTV DNA in larynx from the infection group at 3 dpi was 1.51×10^8^ copies/g, which were 10–100 folds higher than other tissues at the same time. However, the level of the DNA was decreased to 3.27×10^6^ copies/g 7 dpi. Previous reports showed that the ILTV in trachea could be recovered 3–4 dpi and was markedly decreased by day 6 post inoculation [21], [26], [27], which was concordant with the results in this study. In the early stages of infection in chickens, the levels of ILTV DNA in larynx and lung were relatively higher than those in other tissues, which was similar to that reported by Tripathy (1998). The peak of ILTV DNA in the trachea coincided with the severe upper respiratory symptoms. The level of ILTV DNA in larynx of the contact-exposure group peaked at 7 dpi with an average copy number of 3.99×10^5^ copies/g, but the time was delayed and the DNA level was almost the same compared with the infection group as these chickens were infected by contact exposure to the infected chickens.

In previous epidemiological investigations, the laryngeal tracheas were usually collected for virus isolation and identification. When detecting the suspected ILTV infected clinical samples from the field, we found that the ILTV was also detected in the kidney. A lot of chickens with upper respiratory viral disease showed very similar clinical symptoms. Although it has never been reported previously, based on these observations, we decided to analyze the tissue distribution of ILTV in experimentally infected chickens because it would be more convenient for detection of ILTV infected clinical samples if the tissues besides the upper respiratory tract tissues could be used for detection and isolation of ILTV. Interestingly, real-time PCR developed in this study could be used to detect the ILTV DNA in all the collected tissues of the ILTV infected chickens from 1 to 28 dpi. It suggested that all of these tissues tested in this study were suitable for viral detection by using the high sensitive real-time PCR assay, especially the larynx in the early stages of infection. In comparison, ILTVs were detected by immunohistochemical assay in 10 of 17 tissues in the infection group and the contact-exposure group at 3, 7, and 14 dpi. It suggested that the real-time PCR is much more sensitive than the immunohistochemical assay for detection of ILTV in tissues.

The real-time PCR assay developed in this study was applied for the first time to analyses of the distribution of ILTV in the tissues of experimentally infected chickens.It can be utilized as a rapid method to confirm the ILTV diagnosis in the field and is useful for screening clinical samples in epidemiological studies. PCR techniques developed for detection of ILTV so far cannot be used to differentiate the ILTV vaccine strains from field virulent virus strains due to the high similarities of the nucleotide sequences between them. The most effective molecular method for ILTV differentiation is PCR followed by restriction fragment length polymorphism, but even restriction fragment length polymorphism could not differentiate all the tested ILTV vaccine strains from the field strains [23], [28]–[31]. Therefore PCR and real-time PCR techniques are still the very useful and practical techniques for detection of ILTV in the experimental infections and the field cases.

## Conclusion

In this study, a rapid and sensitive real-time PCR assay for the detection and quantitation of ILTV was developed and evaluated. The sensitivity, specificity, and reproducibility of the ILTV real-time PCR assay revealed its suitable application for detection and quantitation of ILTV in the experimentally infected chickens and the clinical cases. The levels of ILTV DNA in the tissues of the chickens infected with ILTV at different times were determined and quantified. The results in this study can help us understand the regular distribution pattern of ILTV *in vivo*.
